# Diminished Expression of P-glycoprotein Using Focused Ultrasound Is Associated With JNK-Dependent Signaling Pathway in Cerebral Blood Vessels

**DOI:** 10.3389/fnins.2019.01350

**Published:** 2019-12-17

**Authors:** HyoJin Choi, Eun-Hee Lee, Mun Han, Sang-Hyun An, Juyoung Park

**Affiliations:** ^1^Medical Device Development Center, Daegu-Gyeongbuk Medical Innovation Foundation, Daegu, South Korea; ^2^Laboratory Animal Center, Daegu-Gyeongbuk Medical Innovation Foundation, Daegu, South Korea

**Keywords:** blood-brain barrier, focused ultrasound, P-glycoprotein, c-Jun N-terminal kinase signaling, vessel integrity

## Abstract

MRI-guided focused ultrasound (MRgFUS) combined with microbubbles (MBs) is a promising technology that can facilitate drug delivery through a temporarily disrupted blood-brain barrier (BBB) and induce the down-regulation of P-glycoprotein (P-gp) expression on the blood vessels. Despite the increasing evidence regarding the down-regulation of P-gp expression after MRgFUS BBB disruption (BBBD), its underlying molecular events remain unclear. The aim of this study was to evaluate the underlying mechanism of FUS BBBD-mediated P-gp down-regulation. While our results showed down-regulation of P-gp at 24 h post-BBBD in transcriptional and translational levels, restoration to the normal expression appeared at different time points for transcriptional (72 h) and translational (120 h) levels. In addition, the signaling molecule, JNK, was significantly activated in the cerebral blood vessels at 24 h post-BBBD. Although P-gp levels were significantly decreased, the expression levels of proteins involved in the integrity of blood vessels, such as Glut1, ZO-1 and occludin, were not decreased at 24 h post-BBBD. Our study suggests that the JNK signaling pathway is involved in the regulation of FUS-induced P-gp expression, without affecting vessel integrity, and a detailed regulatory mechanism can provide the basis for clinical application of FUS to the treatment of neurological disease.

## Introduction

The blood-brain barrier (BBB) is a physical barrier that comprises impermeable brain capillaries, supported by microvascular endothelial cells, pericytes, astrocytes, tight junctions (TJs), and a basal membrane. It plays an essential role in maintaining homeostasis in the nervous system. In addition, the endothelial cells of the BBB have highly selective transporters as functional barriers, limiting free diffusion (Abbott, 2013). However, it presents a major challenge in terms of drug delivery for the treatment of neurological disorders. Recently, focused ultrasound (FUS) technique has emerged as a powerful and feasible transcranial treatment. FUS combined with microbubbles (MBs) is a non-invasive and target-specific drug delivery technique that can enhance drug permeability into the brain by temporarily disrupting the BBB (Burgess et al., 2015). A wide range of substances, such as compounds, cells, viral vectors, and antibodies, have been effectively delivered into specific brain regions using FUS combined with MBs (Jordao et al., 2010; Park et al., 2012; Thevenot et al., 2012; Alkins et al., 2013). In addition, it is reported that the mechanical stress induced to the blood vessel wall by the oscillation of the MBs could induce the suppression of P-glycoprotein (P-gp) expression in the endothelial cells of the BBB (Cho et al., 2016; Aryal et al., 2017). However, the underlying mechanism for the temporary reduction of P-gp expression remains largely elusive.

P-glycoprotein, a member of the ATP binding cassette (ABC) superfamily, is known as one of the most important efflux transporters considered to be a priority in the research for drug delivery development (Sharom et al., 2001; Varma et al., 2003; Sharom, 2011). It can extrude a wide range of structurally and functionally diverse compounds from the brain into the blood. Thus, P-gp can contributes the reduce of drug absorption, permeability and the decrease the drug retention time (Gottesman et al., 2002; Amin, 2013). P-gp has also been found to be overexpressed in tumor cells and the blood-tumor barrier (BTB), and is responsible for the multi-drug resistance in chemotherapy cancer treatment (Bansal et al., 2009; Martins et al., 2010). Previous studies have demonstrated that P-gp inhibition strategies, such as administration of P-gp inhibitors, have a potential role for the enhancement of drug uptake into the target brain region (Schinkel et al., 1994, 1996; Sadeque et al., 2000; Dagenais et al., 2004). Based on these earlier studies, it is believed that inhibition of the P-gp expression or function can improve the effectiveness of chemotherapy. Several signaling pathways, such as MAPK (mitogen-activated protein kinase), JNK (c-Jun NH2-terminal kinase), p38, cAMP-dependent protein kinase, PI3K (phosphatidylinositol 3-kinase), and PKC (protein kinase C) have been suggested to genetically regulate P-gp expression by transcription factors against these signaling pathway (Miller, 2010). In particular, JNK is a well-known stress-responsive kinase that is involved in neuroinflammation, BBB disruption, and oligodendrocyte progenitor apoptosis in the immature brain (Wang et al., 2012). It is reported that JNK signaling also plays a crucial role in suppression of the TJ proteins related with BBB disruption induced by ischemia and subarachnoid hemorrhage (Chen et al., 2012; Wang et al., 2012). However, the mechanism of JNK-mediated P-gp downregulation in BBB disruption induced by FUS is not yet elucidated.

In this study, to better understand the underlying molecular mechanism of FUS-mediated P-gp downregulation, the transcriptional and translational expression levels of P-gp in the cerebral blood vessel were determined at several time-points until 120 h post-BBB disruption (BBBD). We demonstrated that FUS-mediated P-gp downregulation is associated with the JNK signaling pathway. Furthermore, we found that TJ protein expression levels in the BBB-disrupted blood vessels were not decreased 24 h after FUS, suggesting that the JNK signaling can be attributed to distinct pathways that differ from those regulating the TJ proteins. This study illustrates a novel underlying mechanism for FUS-mediated P-gp regulation that requires phosphorylated JNK (pJNK), induced by FUS, for the downregulation of P-gp expression. This could provide further insight for the clinical application of FUS in the development of an efficient and safe treatment of neurological diseases.

## Materials and Methods

### Animals

Seven-week-old, male Sprague-Dawley rats (∼250 g) were used for the experiments. The animals were obtained from Orient Bio Inc., (Seongnam, South Korea). Animals were anesthetized using a mixture of Zoletil 25 mg/kg (Virbac, Carros, France) and Rompun 4.6 mg/kg (Bayer, Leverkusen, Germany) that was administered intramuscularly, and were constantly monitored throughout the course of the experiment. There was no evidence of pain or suffering as a result of the procedure. All experiments were performed in line with the ethical guidelines of the Daegu-Gyeongbuk Medical Innovation Foundation (DGMIF) and were approved by the Institutional Animal Care and Use Committee (IACUC, DGMIF-15122903-10).

### Blood-Brain Barrier Disruption System

Preclinical MRI-guided focused ultrasound (MRgFUS) system (RK-100, FUS instruments, Toronto, Canada) was used for BBB disruption. The system is composed of an air-backed, single-element, spherically curved, piezoelectric transducer (FUS Instruments, Toronto, ON, Canada) with a diameter of 75 mm and radius of curvature of 60 mm. The frequency of the transducer resonance was 1 MHz. Ultrasound pressure distribution at the focal region on the free field was measured using an Acoustic Intensity Measurement System (AIMS III, ONDA, Sunnyvale, CA, United States) with a hydrophone (HGL-400, ONDA, Sunnyvale, CA, United States). The transducer was submerged in a water tank filled with degassed water and the animal was placed on an MR-compatible animal bed with its head submerged in the water. Then, the preclinical MRI images that would provide guidance for the target brain region were transferred to the FUS system and the coordinates between the two systems were synchronized.

MR images were utilized as an image guidance for the focused ultrasound system. A radio frequency coil with an inner diameter of 86 mm was used for transmission of the signal. Two-dimensional relaxation enhancement (2D RARE) pulse sequence was applied for the acquisition of T1-weighted images, with the parameters set as follows: field view: 40 × 40 mm^2^, matrix size: 256 × 256 mm^2^, axial and coronal slices: 1.5 mm thickness without gap, repetition time (TR): 1500 ms, Echo time (TE): 6.5 ms, and number of acquisitions (NEX): 3. For the acquisition of the T2-weighted images, all parameters were same as for the T1-weighted images, except for the following: TR: 2500 ms, TE: 33 ms, and NEX: 2. The T2-weighted images were used for coordination with the MRgFUS system.

### BBBD Procedure

The hair on the heads of anesthetized rats were removed using a shaving razor and hair removal cream. Rats were placed on an MR-compatible bed. 2D RARE pulse sequence was used for the acquisition of T1- and T2-weighted images, which were used as an image guide. Depending on the amount of BBBD tissue necessary for analysis, different areas were targeted with FUS using the T2-weighted images of the rat brain as guidance. For the molecular-based analysis, the whole thalamus region was targeted because a larger quantity of tissue was necessary, whereas for the histological samples, we aimed for a part of the caudate putamen and the thalamus area for comparison. Prior to the FUS application, activated microbubbles (0.02 ml/kg, Definity, Lantheus Medical Imaging, North Billerica, MA, United States) were diluted 1:50 in normal saline and injected through a tail vein catheter using an automated syringe pump 11 (Harvard Apparatus, Holliston, MA, United States) for 10 s as an initiation. The peak negative acoustic pressure of 0.5 MPa at the above −3 dB focal region was used for the experiment. The ultrasound pressure distribution at the focal region on the free field was measured using an Acoustic Intensity Measurement System with a hydrophone (HGL-400, ONDA, Sunnyvale, CA, United States). Subsequently, 0.5 MPa peak negative acoustic pressure was applied over the target focal region to disrupt the BBB, with the micro-infusion lasting over 120 s.

After the MRgFUS, T1-weighted MR images were acquired again using 0.2 mM/kg gadolinium-based contrast agent (Gd-DTPA) (Magnevist^®^, Bayer HealthCare Pharmaceuticals, Berlin, Germany) to confirm that the BBB permeabilized. To determine the disrupted BBB regions, 500 μl of Evans Blue (Sigma-Aldrich, St. Louise, MO, United States) was injected intravenously. All brains were perfused and fixed through transcardial perfusion (0.9% NaCl, 100 mL; 4% Buffered formalin phosphate, 250 mL) at different time points.

### Purification of Rat Brain Vessels

Rat brain blood vessels were isolated from the thalamus region after removing the choroid plexuses, following previously described methods (McNeill et al., 1999; Placido et al., 2017). In brief, the extracted brain was homogenized in ice-cold Hanks balanced salt solution (HBSS, 137 mM Sodium chloride, 5 mM potassium chloride, 4.1 mM Sodium bicarbonate, 1.3 mM Calcium chloride, 0.5 mM Magnesium chloride, 0.4 mM Magnesium sulfate, 0.4 mM Potassium phosphate monobasic, 0.3 mM Sodium Phosphate dibasic, 5.5 mM Glucose, pH 7.4). After centrifugation at 2000 *g* for 10 min, the pellet was resuspended in ice-cold HBSS and layered over with 16% dextran solution (Sigma-Aldrich, St. Louis, MO, United States), followed by centrifugation at 4400 *g* for 15 min. The procedure was repeated twice to collect the top and middle layers containing the blood vessels, which were then filtered through a 20 μm nylon mesh. The vessels on the top of the nylon mesh were used for detection of RNA and protein expression levels of P-gp.

### Real-Time Quantitative PCR (qRT-PCR)

Total RNA samples were extracted from the brain vessels using RNAiso Plus reagent (Takara Bio Inc., Otsu, Shiga, Japan) according to the manufacturer’s instructions. The total RNA (1 μg) from each sample was reverse-transcribed into cDNA using Primescript^TM^ 1st strand cDNA synthesis kit (Takara Bio Inc., Otsu, Shiga, Japan) using C1000 Touch^TM^ Thermal Cycler (Bio-Rad, Hercules, CA, United States). The levels of gene expression were quantified by real-time PCR using SYBR^®^ *Premix Ex Tag*^TM^ II (Tli RNaseH Plus, Takara Bio Inc., Otsu, Shiga, Japan) and LightCycler^®^ 480 II Real time PCR instrument (Roche, Mannheim, BW, Germany). The sequences of the primers were as follows: P-gp: forward, 5′–ACAGAGGATCGCC ATTGCCC–3′, and reverse, 5′–TGGTGGTCCGGCCTTCT CTA–3′, (GenBank: NM_133401), and β-actin: forward, 5′–CAC GATGGAGGGGCCGGACTCATC–3′, and reverse: 5′–TAAA GACCTCTATGCCAACACAGT–3′) (GenBank: NM_031144). The β-actin gene was amplified separately as an internal control to normalize for P-gp expression using the LightCycler^®^ software (Roche, Mannheim, BW, Germany).

### Immunoblot

The rat brain vessels were lysed in radioimmunoprecipitation assay buffer containing protease inhibitor (150 mM Sodium chloride, 0.1% SDS, 1% Triton X-100, 1% Sodium deoxycholate, and 50 mM Tris–Hcl [pH 7.5, 2 mM EDTA]). After centrifugation at 16000 *g* for 20 min, the protein concentration was determined using Pierce^TM^ BCA Protein Assay Kit (Thermo Fisher Scientific, Waltham, MA, United States). The proteins (30 μg/lane) were separated using a 10% sodium dodecyl sulfate polyacrylamide gel electrophoresis and were transferred onto a polyvinylidene difluoride membrane (Millipore, Billerica, MA, United States). Membranes were blocked with 5% non-fat skim milk in phosphate buffered saline containing 0.05% tween 20 for 1 h at room temperature (RT) and were incubated overnight at 4°C with rabbit monoclonal anti-P-gp (Abcam, Cambridge, MA, United States) or anti-β-actin antibodies (Abcam, Cambridge, MA, United States), followed by incubation with horseradish peroxidase (HRP)-conjugated anti-rabbit IgG secondary antibody (Abcam, Cambridge, MA, United States) for 2 h at RT. The signal was detected using an ECL plus chemiluminescence kit (Amersham Pharmacia Biotech Inc., Piscataway, NJ, United States). The band density was quantified by ImageJ software (1.52v, National Institutes of Health, Bethesda, MD, United States) (Yang and Rosenberg, 2011).

### Immunohistochemistry

Immunohistochemistry was performed according to a previously described method (Cho et al., 2016). Rats were sacrificed and transcardially perfused with 0.9% NaCl. Brains were post-fixed overnight in ice-cold 4% formaldehyde. Fixed brains were cryopreserved through 10, 20, and 30% sucrose gradients for dehydration. The frozen brain tissue was cut in 50-μm thick slices. Immunolabeling for P-gp was performed 24 and 120 h after BBBD, whereas the pJNK and TJ proteins were stained in 24 h post-BBBD. Specific primary antibodies included rabbit monoclonal anti-P-glycoprotein, mouse monoclonal anti-GLUT1 (Abcam, Cambridge, MA, United States), rabbit polyclonal anti-pJNK (Cell signaling, Danvers, MA, United States), rabbit polyclonal anti-ZO-1, and rabbit polyclonal anti-Occludin (Invitrogen, Carlsbad, CA, United States). HRP-conjugated secondary antibodies included Alexa Fluor 488 or 546 goat anti-rabbit IgG, and Alexa Fluor 488 or 546 goat anti-mouse IgG (Abcam, Cambridge, MA, United States). The slides were mounted with fluorescence mounting medium (Dako, Glostrup, Denmark).

For the histological analysis, the brain was immediately immersed in 10% formaldehyde and fixed for 1 week. The fixed brain was then serially sectioned as 5 μm slices in axial plane, which were stained with hematoxylin and eosin (H&E) every 50th section (250 μm apart).

### Image Analysis

The tissue slides from immunofluorescence and histology were scanned by the Pannoramic Scan II (3DHistech, Budapest, Hungary). The acquired images were processed using the CaseViewer software (2.1v, 3DHistech, Budapest, Hungary). Rectangular regions of interest (ROIs) were outlined in the BBBD region and in a control region matched using the MR images. Fluorescence intensity was defined as the relative change in fluorescence compared to that of the control, which was normalized to 100% using ImageJ software.

### Statistical Analysis

The statistical significance was analyzed by comparing the results obtained from the BBBD region with those obtained in the control region, using two-tailed unpaired Student’s *t*-tests. Pearson’s product-moment correlation coefficient (Pearson’s correlation) was used for correlation analysis between P-gp protein expression levels and their coding mRNA expression. The *P-*value below 0.05 was defined as statistically significant. All statistical analyses were performed using SPSS software (version 22.0, SPSS Inc., Chicago, IL, United States).

## Results

### Confirmation of MRgFUS-Induced BBBD

**FIGURE 1:**
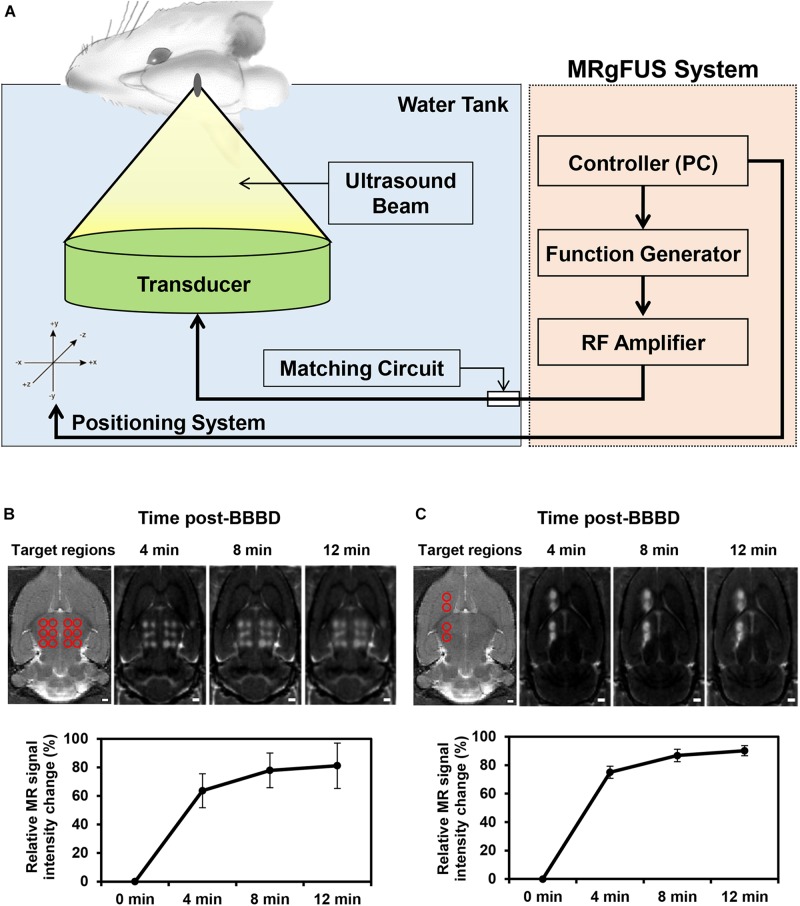
Schematic of the MRgFUS system used for BBBD and changes of BBB permeability after BBBD. **(A)** Rats were placed in supine position and FUS waves generated upward to focal region in the brain. **(B)** To analyze the mRNA/protein expression level of P-gp, the 12 targeted points of the thalamus region were represented in a T2-weight MR image (top, left panel). Contrast-enhanced T1-weighted MR images were represented at three-time. The graph reflected mean intensity of contrast enhancement compare with pre-contrast T1-weighted MR images (bottom). **(C)** For immunohistochemistry analysis, T2-weighted MR Image of four targeted points was represented in caudate putamen and thalamus (top, left panel). Contrast-enhanced T1-weighted MR images reflected the increase in intensity according to continuous time. The relative signal intensity changes were obtained by Contrast-enhanced T1-weighted MRI (bottom).

A schematic diagram depicting the BBBD induced by MRgFUS is presented in Figure 1A. Depending on the purpose of the experiment, to detect the molecular expression levels using qRT-PCR/immunoblot analysis or immunohistochemistry analysis, the MRgFUS was applied to 12 targets in the whole thalamus region or to two targets in the caudate putamen and thalamus region, respectively, (Figures 1B,C). To obtain sufficient amount of mRNA and protein after isolation of the blood vessels in the targeted BBBD region, we used a BBB disruption model by FUS stimulation in the 12 target points of the brain region (Figure 1B). In addition, the left hemisphere was also targeted for BBBD so that a comparison could be made with the contralateral region for the immunofluorescence staining (Figure 1C). BBBD was assessed using contrast-enhanced T1-weighted images after Gd-DTPA administration in a time-dependent manner. The increased BBB permeability in the FUS target regions was evident from the progressive signal enhancement over-time. The mean intensities over-time were used to plot the curve normalized by pre-contrast T1-weighted images. The signal enhancement of the whole thalamus-targeted regions was increased from 63.6 ± 13.3 to 81.2 ± 17.8% (Figure 1B), and another targeting group showed similar intensity changes, from 75.1 ± 5.3% to 90.2 ± 4.4% (Figure 1C). These results (Figures 1B,C) demonstrated that the FUS treatment similarly increased the BBB permeability; thus, we were able to conduct P-gp qRT-PCR/immunoblot and immunohistochemistry analysis. In addition, FUS-BBBD was confirmed by the fluorescence image of Evans blue dye (Supplementary Figure S1).

### The Changes of P-gp Expression at Different Time Points After BBB Disruption

**FIGURE 2:**
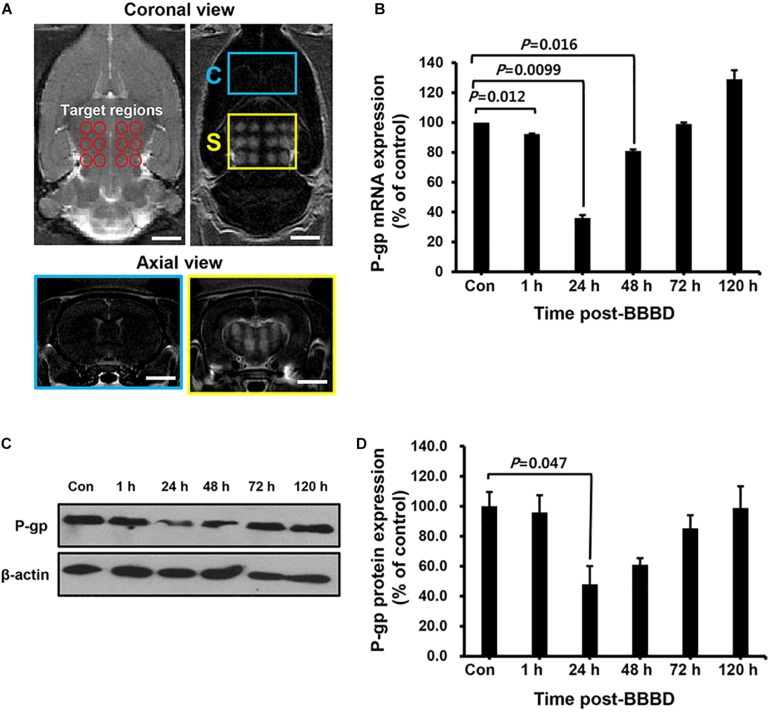
The expression level of P-gp after BBBD by MRgFUS in a time-dependent manner. **(A)** The coronal view of the targeted region (top, left panel) and BBB disrupted region (top, right panel) in T2- and T1-weighted images, respectively. The axial views of the brain indicated Gd-DTPA distribution in correspondence with coronal views of the control region (bottom, blue box) and BBBD region (bottom, yellow box) after BBBD. **(B)** Relative P-gp mRNA expression was measured by qRT-PCR. The bar graph represented the percentage of P-gp expression level, and calculated by normalizing with control and using beta-actin as internal control gene. **(C)** Immunoblot was performed for P-gp and beta-actin from cerebral blood vessel after BBBD in a time-dependent manner. **(D)** The protein levels from Immunoblot were calculated by densitometric analysis and normalized with beta-actin. Results were presented as the mean ± SEM (*n* = 3).

To elucidate the transcriptional/translational levels of P-gp, the cerebral blood vessels were isolated for qRT-PCR and immunoblot at different time points after BBBD. All targeted regions showed a similar signal enhancement in the BBBD regions (Figure 2A). As shown in Figure 2B, the mRNA expression level of P-gp was decreased compared to that of the control by 92.3 ± 0.3% (*P* = 0.012) and 36.0 ± 2% (*P* = 0.0099) at 1 and 24 h post-BBBD, respectively. At 48 h post-BBBD, the P-gp mRNA level was increased by 81.2 ± 1% (*P* = 0.016). It restored to normal levels 99.0 ± 1% (*P* = 0.25) at 72 h post-BBBD, and continuously elevated to 129.5 ± 6% (*P* = 0.064) at 120 h post-BBBD (Figure 2B). To determine the relation between P-gp mRNA and protein expression, immunoblot analysis was performed at the same time points (Figures 2C,D). Similar to the transcriptional expression patterns, the translational level of P-gp showed the greatest decrease of approximately 48 ± 12.2% (*P* = 0.047) at 24 h post-BBBD. The tendency of decrease in P-gp translational expression was sustained until 48 h, when it reached 61 ± 3.8% (*P* = 0.117). The P-gp protein expression was recovered to normal level 98.9 ± 10.2% (*P* = 0.48) after 120 h post-BBBD. These results suggested that BBBD induced by MRgFUS could transiently regulate the P-gp expression in the cerebral blood vessels.

### The Relation of P-gp Transcriptional and Translational Expression in Cerebral Blood Vessels After BBBD

**FIGURE 3:**
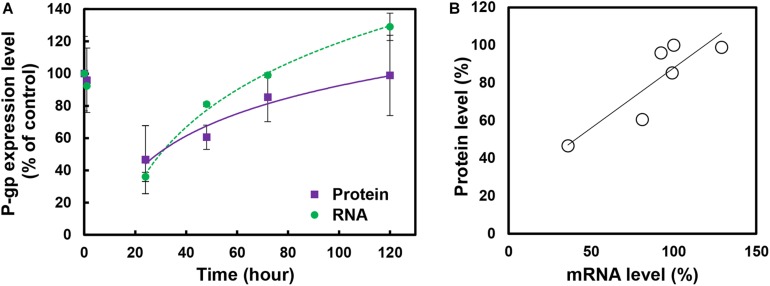
Kinetics and correlation of P-gp expression in cerebral blood vessel after BBBD. **(A)** The curve plot showed changes between RNA and protein expression level at different times post-BBBD. **(B)** Scatter plot of P-gp protein expression level across its RNA expression. Correlation was assessed by Pearson’s correlation as positive linear regression (*r*^2^ = 0.76, *P* < 0.01).

To examine the kinetics of P-gp expression induced by BBBD, a curve plot was constructed using the data of qRT-PCR and immunoblot against different time points post-BBBD (Figure 3A). P-gp transcriptional and translational expression levels were sharply decreased until 24 h post-BBBD and then gradually elevated to the control level from 48 to 120 h post-BBBD. To evaluate the correlations between the transcriptional and translational expression, relative P-gp expression levels were plotted using linear regression (Figure 3B). It was observed that a significant positive correlation existed between P-gp transcriptional and translational expression levels (*r*^2^ = 0.76, *P* < 0.01, Pearson’s correlation). These results indicated that the P-gp expression in the vascular endothelium was affected by transcriptional regulation induced by BBBD, suggesting that the transcriptional regulation of P-gp expression caused by the FUS stimulation may be the cause of the correlation with the translational expression level.

### Evaluation of P-gp Expression After BBBD With Immunofluorescence Staining

**FIGURE 4:**
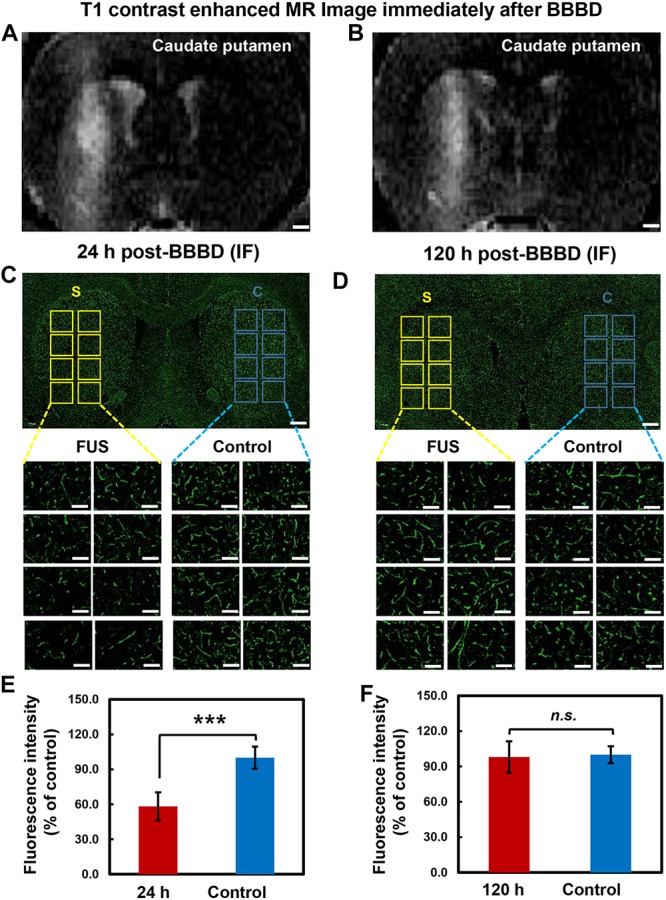
Immunolabeling of P-gp in brain tissue after BBBD. Representative post-sonication T1-weighted MR images of animals in **(A)** group 1 (scarified at 24 h post-BBBD), **(B)** group 2 (scarified at 120 h post-BBBD). **(C,D)** P-gp immunostaining was performed in brain tissue at 24 h post-BBBD **(C)** and 120 h post-BBBD **(D)**. Each 8 ROIs were selected for BBBD region (S, yellow square box) and control region (C, blue square box). The selected ROIs were magnified 20 times (original magnification, ×20) **(E,F)** The intensity of P-gp expression were measured and converted to relative intensity compared with control. It was presented as the mean ± standard deviation (SD) (*n* = 8, ^∗∗∗^*P* < 0.001, n.s.; no significance) Scale bar was represented 1 mm in **(A,B)**, or 50 μm in **(C,D)**.

To determine the temporal relation of P-gp expression in the cerebral blood vessels, different brain tissues were stained with P-gp antibody at 24 h post BBBD (Figures 4A,C,E) and 120 h post-BBBD (Figures 4B,D,F), respectively. Based on the result from the qRT-PCR and immunoblot analysis, we determined the optimal time points for immunofluorescence analysis to detect the largest decrease at 24 h and the complete recovery of P-gp expression at 120 h. Each of the eight ROI areas (Figures 4C,D) in the caudate putamen region was selected by matching the BBBD regions from the T1-weighted MR images (Figures 4A,B) with the FUS regions, and was compared with the contralateral regions. In the FUS-treated hemisphere, P-gp expression was significantly decreased to 58.2 ± 12.1% (*P* < 0.001) at 24 h post-BBBD and completely recovered at 98.0 ± 13.4% (*P* = 0.37) compared to that in the contralateral hemisphere at 120 h post-BBBD (Figures 4E,F). The results of the blood vessel staining for P-gp coincided with the expression patterns of transcriptional and translational levels. Our results revealed a real *in situ* P-gp expression in the blood vessels located mainly in the FUS-stimulated area, suggesting its relation to the transcriptional and translational expression levels.

### JNK Activation in the Vascular Endothelium of the FUS Targeted Regions

**FIGURE 5:**
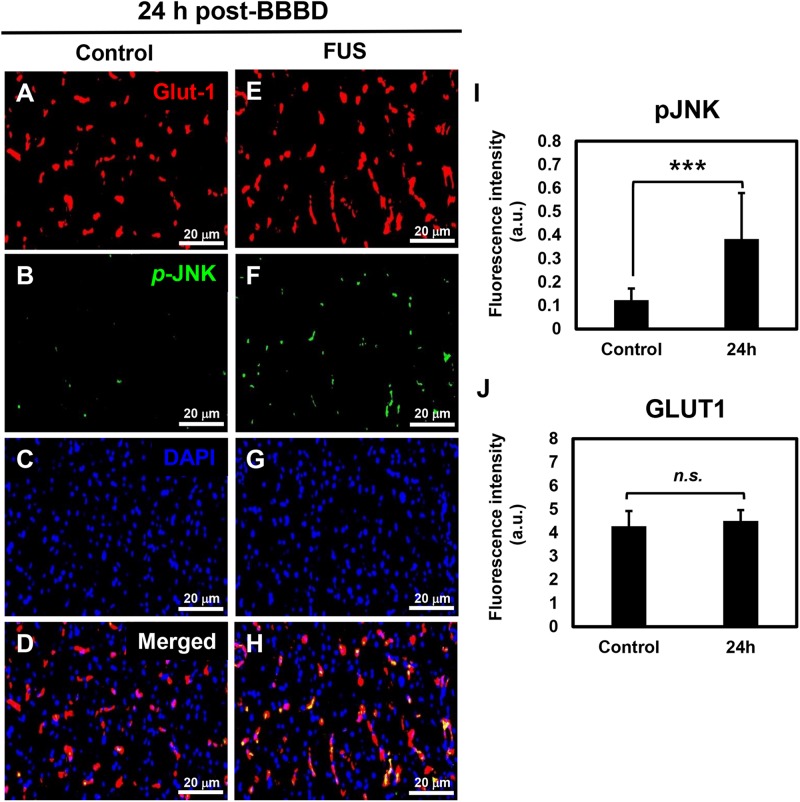
JNK activation in vascular endothelium at 24 h post-BBBD. Immunofluorescence images were collected from the control hemisphere **(A–D)** and FUS **(E–H)** hemispheres at 24 h post-BBBD. Glut1 (red) was used as a vascular endothelial marker. The fluorescence intensities were calculated from 15 ROIs per hemisphere in three sections **(I,J)**. Data are presented as the mean ± SD, ^∗∗∗^*P* < 0.001). Scale bar was represented 20 μm.

It was reported that the JNK signaling pathway was involved in direct regulation of P-gp gene expression in cancer cells (Zhou et al., 2006). To investigate the relation between the P-gp downregulation and JNK activation in the vascular region, immunofluorescence-based double staining was performed with pJNK and Glut1 antibodies. Glut1 was used as a marker to visualize and quantify the vascular endothelial cells (Zeller et al., 1997). The results showed that JNK phosphorylation was significantly increased at the FUS-stimulated hemisphere 24 h after BBBD (Figures 5B,F,I). The expression of pJNK clearly overlapped with the expression of Glut1, known as vascular endothelial cell marker (Figures 5D,H). No differences in vessel density or morphological changes were observed when compared with the control hemisphere (Figures 5A,E,J). DAPI staining was carried out to determine the cell density (Figures 5C,G). These finding suggested that the JNK signaling pathway might be involved in the downregulation of the P-gp in the BBB, which was disrupted by FUS combined with MBs.

### Assessment of Safety

**FIGURE 6:**
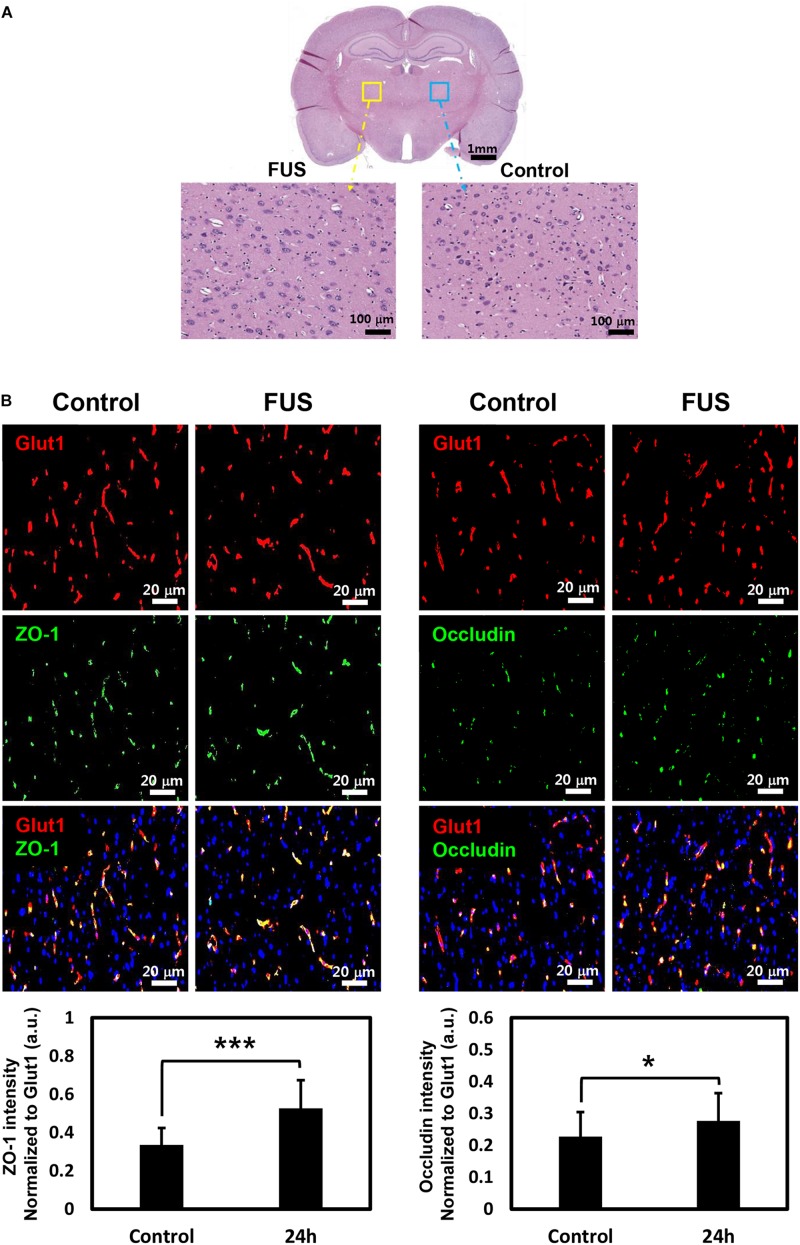
Histology and immunostaining of tight junction (TJ) proteins. **(A)** Whole brain section was stained with H&E at 4 h post-BBBD (top). Left enlarged image showed BBBD region (yellow square box) and right enlarged image showed control region (blue square box) **(B)** TJ protein, ZO-1 (green, left panel), and occludin (green, right panel) was double-stained with vascular endothelial cell marker, Glut1 (red) at 24 h post-BBBD. The fluorescence intensities of ZO-1 (bottom, left) and occludin (bottom, right) were normalized to Glut1 fluorescent. It was presented as the mean ± SD (multiple ROIs (*n* = 30) per hemisphere, ^∗^*P* < 0.05, ^∗∗∗^*P* < 0.001).

Since FUS-induced BBBD was reported to last approximately 4–6 h (Sheikov et al., 2008), H&E staining was performed at 4 h after FUS to confirm tissue damage caused by BBBD. As shown in Figure 6A, no tissue damages or microhemorrhage was observed in the FUS-stimulated hemisphere. In addition, 24 h post-BBBD, we also investigated whether the MRgFUS parameters could affect the expression of occludin and ZO-1, which plays an important role in keeping the BBB impermeable. There was no decrease in the occludin and ZO-1 expression levels after the FUS stimulation (Figure 6B). These results demonstrated that the MRgFUS parameters used in this study were optimal and safe for specific modulation of P-gp without vessel loss or permanent damage of BBB integrity.

## Discussion

In this study, we found that MRgFUS temporarily reduced the P-gp expression through transcriptional regulation. While the MRgFUS stimulation specifically downregulated the P-gp expression, the expression levels of constitutive Glut1, ZO-1 and occludin in the blood vessels were not decreased 24 h after BBBD. To the best of our knowledge, this is the first report of a regulation mechanism of P-gp following MRgFUS combined with microbubbles in cerebral blood vessels. Although further studies are needed to elucidate the transcription factors associated with the regulatory mechanism, our present observation provides the first evidence that FUS affects the P-gp transcriptional and translational regulation via the pJNK signaling pathway.

P-glycoprotein expression has been observed in many types of cells, including the kidney, liver, cells of the gastrointestinal tract and the brain (Davis et al., 2014). In particular, P-gp contributes to the poor success rate of CNS-targeted drugs by limiting the permeability of the BBB. To overcome the difficulties associated with P-gp in drug delivery, many inhibitors were developed, such as verapamil, cyclosporine A, and *trans-*flupenthixol. However, the usage of these inhibitors is limited due to low selectivity and toxicity (Amin, 2013). In order to investigate the expression level of P-gp, we selected time points at 1, 24, 48, 72, and 120 h. Our data represents the tendency of transients to recover from the reduction of P-gp by FUS-BBBD. However, we were limited in our ability to fully understand P-gp kinetics due to missed time points. Although further detailed experiments covering the intermediate time points between 1 and 24 h or 120 h are required, it seems that the increase of P-gp mRNA at 120 h post-BBBD or the immediate change of P-gp mRNA within 1 h could be interpreted as a biological recovery process required to maintain homeostasis in response to mechanical stress induced by FUS. In this study, we demonstrated that MRgFUS has the potential for selective P-gp inhibition, because it did not cause a decrease to constitutive Glut1, TJ proteins in vascular endothelium (Figures 5J, 6B). Furthermore, the results suggest that MRgFUS may be used as a therapeutic tool for P-gp modulation in drug-resistant tissue. In particular, P-gp is highly expressed in multidrug resistant tumors, including malignant brain tumors. It was reported that abnormally developed tumor vessels express P-gp at the same level as normal tissue (Regina et al., 2001). Further extensive experiments using disease models are required to support our hypothesis since the present results are limited to normal animal models. Nonetheless, it is considered that transient modulation of P-gp expression using MRgFUS may provide the benefit of improving the outcome of drug treatment in patients with brain tumors. This technique can also provide an opportunity to rediscover the value of failed drug candidates due to their low permeability, particularly CNS-targeting drugs.

The level of expression between the functional protein and its coding transcript is dynamic. Quantitative study can be used to provide basic information on the multiple processes beyond the transcript concentration to establish protein expression level (Liu et al., 2016). Our results showed that there was a positive correlation between the transcriptional and translational expression of P-gp affected by MRgFUS (Figures 2, 3). This means that MRgFUS can trigger the signal pathways that respond to mechanical stress induced by the microbubble oscillation.

Previous studies have suggested multiple signal pathways that regulate the expression and activity of P-gp in response to xenobiotics, stress and disease (Labialle et al., 2002; Miller, 2010). JNKs are representative stress-activated kinases that can induce P-gp downregulation through catalytic activity by mediating transcription factors (Zhou et al., 2006). Our results suggested that FUS-BBBD-mediated downregulation of P-gp expression is associated with the pJNK signaling pathway without affecting vessel integrity. However, previous studies have reported that pJNK is not associated with the response to mechanical stress following FUS-BBBD (Jalali et al., 2010; Kovacs et al., 2017). The previous studies used western blot analysis to analyze total protein extracts from the parenchymal region after BBBD. In our study, pJNK was detected only in the blood vessel by immunofluorescence analysis. Based on these results, we speculate that pJNK was probably activated in endothelial cells in the vascular region by FUS-BBBD without affecting the neuron in the parenchyma. The pJNK may be related to recovery in the temporarily disrupted vascular environment. Several studies reported that JNK is a positive regulator of angiogenic potential in endothelial cells (Uchida et al., 2008; Ramo et al., 2016). Recently, Hynynen group has also determined an initiation of angiogenic processes 24 h after FUS in transcriptome analysis, and observed angiogenic effects 7 and 14 days after FUS by confirming the relative area of Glut1 immunodetection and BrdU positive endothelial cells density (McMahon et al., 2017, 2018). The results of our study may provide insight into a novel mechanism in which JNK signaling pathway might play a role in the process of angiogenesis.

The effects of FUS could be influenced by diverse factors, including ultrasound frequency, acoustic pressure amplitude, pulse repetition frequency, duration, dose, and size of the MBs. According to the study of Aryal et al. (2017), P-gp suppression and tissue damage were closely related to the FUS exposure level. Therefore, it is important to optimize the acoustic pressure based on the balance between bio-effects and damage. In our previous study (Cho et al., 2016), 0.6–0.65 MPa of FUS acoustic pressure were used. P-gp was significantly reduced at 24 h after FUS-BBBD, but mild microhemorrhage was observed at 4 h. According to our results, given the selection of a FUS parameter that would minimize parenchymal damage and without associated micro-hemorrhages, it was speculated that the intensity of 0.5 MPa leads to decreased P-gp specifically without vessel rupture. Although tissue damage and micro-hemorrhage were not detected in the BBB disrupted region by H&E staining, our finding is limited to addressing inflammatory activation. A recent report (Kovacs et al., 2017) has suggested that FUS-BBBD induces a sterile inflammatory response through the NF-kB pathway in the parenchyma region. However, the previous study did not address whether vessel integrity was affected by the cavitation force of acoustic pressure. Therefore, any further investigation into selecting the optimal FUS parameter that would minimize associated micro-hemorrhages and tissue damage should consider both the bioeffects of a FUS-mediated BBB opening procedure in the parenchymal region on the molecular level and changes in vessel integrity. The expression of TJs is closely involved in BBB function such as integrity. In this study, the signal intensity of ZO-1 and occludin was increased in the vascular region after 24 h of BBBD (Figure 6B). One possible explanation of this increase can be compensatory homeostasis for the temporal disintegration of vessels. However, a quantitative comparison of TJs expression remains to be further investigated.

In this study, we verified the potential and safety of FUS by confirming temporary P-gp reduction and complete recovery with time, at the optimal intensity of 0.5 MPa, without tissue or vascular damage. In addition, our results raise the interesting possibility that pJNK signaling pathway serves as the pivot of transcriptional regulation in the P-gp expression induced by FUS stimulation. These findings will be useful basic information for clinical applications targeting diseases requiring repeated drug administration.

## Data Availability Statement

All datasets generated for this study are included in the article/Supplementary Material.

## Ethics Statement

The animal study was reviewed and approved by the Daegu-Gyeongbuk Medical Innovation Foundation (DGMIF) Institutional Animal Care and Use Committee (IACUC).

## Author Contributions

HC, E-HL, and JP conceptualized and designed the experiments. HC performed immunofluorescence staining and drafted the manuscript. MH carried out MRgFUS experiments. S-HA performed qRT-PCR and immunoblot. E-HL and JP edited and revised the manuscript.

## Conflict of Interest

The authors declare that the research was conducted in the absence of any commercial or financial relationships that could be construed as a potential conflict of interest.

## Supplementary Material

FIGURE S1 The fluorescence image of Evans blue dye at 24 h after BBBD. **(A)** An axial view of T1-weighted image was obtained immediately after BBBD. **(B)** Evans blue was injected immediately after FUS and detected by fluorescence at 24 h post-BBBD (right).
