# Supplementary figures and images for: Diminished Expression of P-glycoprotein Using Focused Ultrasound Is Associated With JNK-Dependent Signaling Pathway in Cerebral Blood Vessels
Source: Front Neurosci. 2019 Dec 17;13:1350. doi: 10.3389/fnins.2019.01350 (PMC6928061; doi:10.3389/fnins.2019.01350)

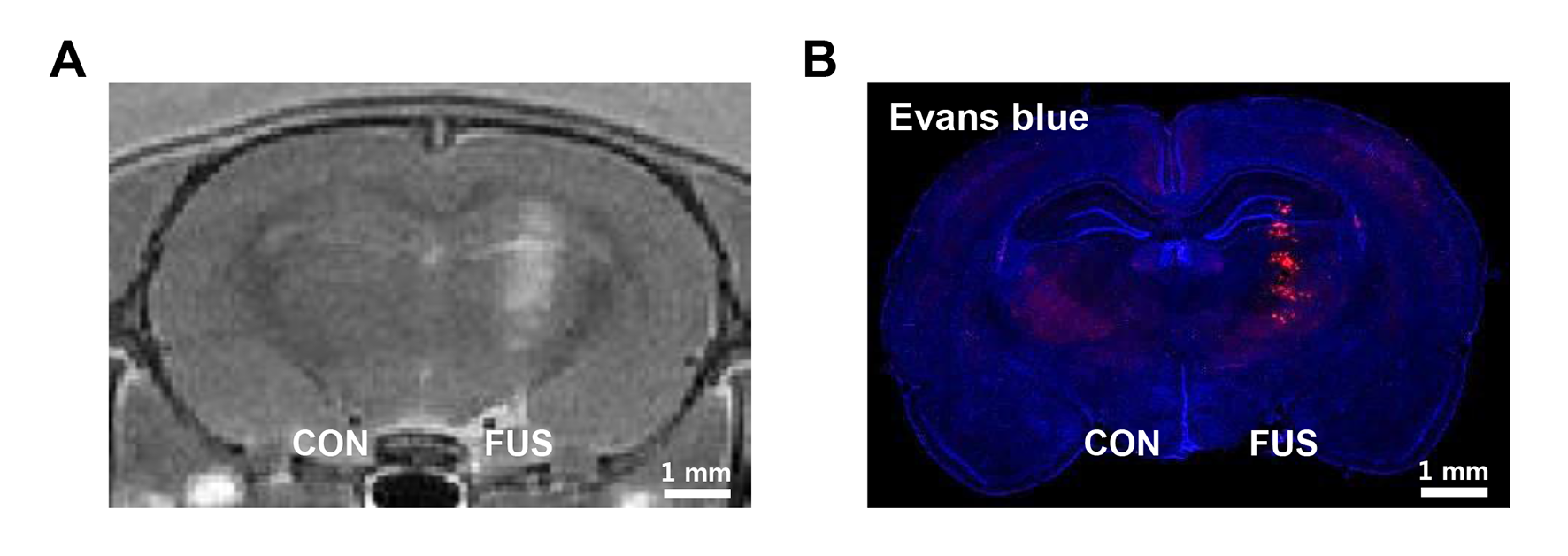

Supplement: FIGURE S1 — The fluorescence image of Evans blue dye at 24 h after BBBD. (A) An axial view of T1-weighted image was obtained immediately after BBBD. (B) Evans blue was injected immediately after FUS and detected by fluorescence at 24 h post-BBBD (right). [file Image_1.TIF]
